# Prevalence and determinants of polypharmacy in Switzerland: data from the CoLaus study

**DOI:** 10.1186/s12913-017-2793-z

**Published:** 2017-12-21

**Authors:** Julien Castioni, Pedro Marques-Vidal, Nazanin Abolhassani, Peter Vollenweider, Gérard Waeber

**Affiliations:** Department of Medicine, Internal Medicine, Lausanne university hospital, Rue du Bugnon 46, 1011 Lausanne, Switzerland

**Keywords:** Polypharmacy, Socio-economic, Smoking, Epidemiology

## Abstract

**Background:**

Polypharmacy is a frequent condition, but its prevalence and determinants in the Swiss mid-aged population are unknown. We aimed to evaluate the prevalence and determinants of polypharmacy in a large Swiss mid-aged population-based sample.

**Methods:**

Data from 4938 participants of the CoLaus study (53% women, age range 40–81 years) were collected between 2009 and 2012. Polypharmacy was defined by the regular use of five or more drugs.

**Results:**

Polypharmacy was reported by 580 participants [11.8%, 95% confidence interval (10.9; 12.6)]. Participants on polypharmacy were significantly older (mean ± standard deviation: 66.0 ± 9.1 vs. 56.6 ± 10.1 years), more frequently obese (35.9% vs. 14.7%), of lower education (66.6% vs. 50.7%) and former smokers (46.7% vs. 36.4%) than participants not on polypharmacy. These findings were confirmed by multivariate analysis: odds ratio and (95% confidence interval) for age groups 50–64 and 65–81 relative to 40–49 years: 2.90 (2.04; 4.12) and 10.3 (7.26; 14.5), respectively, p for trend < 0.001; for low relative to high education: 1.56 (1.17; 2.07); for overweight and obese relative to normal weight participants: 2.09 (1.65; 2.66) and 4.38 (3.39; 5.66), respectively, p for trend < 0.001; for former and current relative to never smokers: 1.42 (1.14, 1.75) and 1.63 (1.25, 2.12), respectively, p for trend < 0.001.

**Conclusion:**

One out of nine participants of our sample is on polypharmacy. Increasing age, body mass index, smoking and lower education independently increase the likelihood of being on polypharmacy.

**Electronic supplementary material:**

The online version of this article (10.1186/s12913-017-2793-z) contains supplementary material, which is available to authorized users.

## Background

In industrialized countries, population ageing is paralleled by an increase in the number of drugs prescribed [1, 2]. An increasing number of subjects are with polypharmacy, defined as the regular intake of five or more medicines [1–3]. The prevalence of polypharmacy is believed to have doubled during the last decade [2, 3]. Still, the reported prevalence rates vary considerably, from 10% in a study conducted in 2006 in the Greek general population [4] to 75% in a study conducted in 2012 in the Austrian nurse care system [5].

Several factors have been shown to be associated with polypharmacy. Ageing and its associated multi-morbidities are the most important [6–9]. The prevalence of polypharmacy can be as high as 60% in the general population aged over 65 years [10, 11], although high prevalence rates have also been reported for younger people [2, 3, 12, 13]. Indeed, two thirds of all individuals with polypharmacy are under 70 years old [14]. Current and/or former smoking and obesity have also been positively associated with polypharmacy [4, 15], while the effect of education is controversial, as higher levels of polypharmacy have been reported among high [4] or low [6, 16] educational groups, while even other studies found no differences between educational groups [17].

Polypharmacy increases the probability of drug-drug interactions and adverse drug reactions [3] and is associated with a higher risk of falls, hospitalisation, poor functional status, morbidity and mortality [18–20]. Nevertheless, polypharmacy might be necessary and beneficial to the patient if adequately prescribed [21, 22], and only inappropriate polypharmacy should be reduced [23].

From a clinical and public health perspective, it is important to evaluate the prevalence of polypharmacy in the mid-aged population, as it is a marker of multimorbidity and of potential adverse drug reactions. Further, with the exception of a study which focused on polypharmacy using health reimbursement claims [12, 24], no information is available regarding the prevalence and determinants of polypharmacy in the Swiss mid-aged population.

Our study aimed to: 1) assess the prevalence of polypharmacy in the Swiss mid-aged population; and 2) identify the individual and socio-economic factors associated with polypharmacy. We also assessed the type of drugs most commonly prescribed to patients with polypharmacy.

## Methods

### Study population and design

The Colaus study (www.colaus.ch) is an ongoing prospective survey investigating the biological and genetic determinants of cardiovascular disease in the population of Lausanne, Switzerland. Detailed descriptions of the study design have been reported elsewhere [25]. A simple, non-stratified random sample of 19,830 subjects (corresponding to 35% of the source population) was drawn [25]. Inclusion criteria were: (a) written informed consent and (b) willingness to take part in the examination and to provide blood samples.

The baseline study was conducted between 2003 and 2006 and included 6733 participants, with a participation rate of 41%; the first follow-up was conducted between April 2009 and September 2012, five and a half year on average after the baseline and included 5064 participants (75.2%) [26].

The baseline evaluation included an interview, a physical exam, blood sampling and a set of questionnaires. All participants were interviewed by trained recruiters regarding personal and family history of cardiovascular disease and risk factors and medicines taken. The questionnaires can be obtained from the authors upon request. The same procedure (questionnaires, interview and physical examination) was applied at follow-up. The data from the first follow-up was used in this study.

### Ethical statement

This study was conducted according to the guidelines laid down in the Declaration of Helsinki and all procedures involving human subjects were approved by the Institutional Ethics Committee of the University of Lausanne (decision reference 33/09). Written informed consent was obtained from all participants.

### Clinical and biological parameters

CVD and medication status were assessed by questionnaire. Smoking status was defined as never, former (irrespective of the time since quitting) and current (irrespective of the amount smoked). Educational level was categorized as low (obligatory school or apprenticeship), medium (high school), or high (university degree). Marital status was dichotomized into living alone (single, divorced, widowed) and living with somebody (married or partnership). Country of birth was categorized into Switzerland and other [25]. Body weight and height were measured with participants standing without shoes in light indoor clothes. Body weight was measured in kilograms to the nearest 100 g using a Seca® scale, which was calibrated regularly. Height was measured to the nearest 5 mm using a Seca® height gauge [25]. Body mass index (BMI) was defined as weight/height2. Overweight was defined as 25 ≤ BMI < 30 kg/m2 and obesity as BMI ≥ 30 kg/m2^.^ Venous blood samples (50 ml) were drawn after an over-night fast, and most clinical chemistry assays were performed by the CHUV Clinical Laboratory on fresh blood samples. Measurements included blood lipids, liver markers, cytokines and adipokines.

### Polypharmacy

Participants were asked to bring all their medicines, which were checked by the research assistants. Participants were asked if the medicines were prescribed by a doctor or obtained over the counter. The regular consumption of the medicines over the last six months was also queried. Posology was not taken into account, i.e. a patient with propranolol 3 × 40 mg a day was considered as taking a single drug.

Polypharmacy was defined as the regular use of five or more different pharmacologically active medicines, regardless if a medicine contained one or more components [21, 27]. Excessive polypharmacy was defined as the regular use of ten or more medicines [6]. Regular use was defined as a medicine taken regularly over the past six months. Only medicines considered as medically needed (i.e. prescribed by a doctor) were considered; hence, we excluded medicines obtained over the counter, alternative therapies such as plant extracts, dietary supplements and homeopathy.

### Exclusion criteria

Participants were excluded if they missed any information regarding individual, clinical or socio-economic data.

### Statistical analysis

Statistical analyses were performed using Stata version 14.1 (Stata Corp, College Station, Texas, USA). Descriptive results were expressed as mean ± standard deviation (SD) for continuous variables or as number of participants (percentage) for categorical variables. For prevalences, exact 95% confidence intervals were also computed. Bivariate analysis was performed using Student’s t-test for continuous variables and chi-square test for categorical variables. Multivariate analysis was performed using logistic regression and results were expressed as multivariate-adjusted odds ratio (OR) and [95% confidence interval (CI)]. Statistical significance was considered for a two-sided test with *p* < 0.05. Two sensitivity analyses were performed: the first one included over the counter (OTC) drugs, and the second considered all active substances among prescribed and OTC drugs. As some drugs combine several pharmacologically different active substances, we identified the ATC codes corresponding to combinations of different active substances (Additional file 1 **:** Table S1), excluding combinations of vitamins and minerals (ATC codes A11A; A11C; A11D; A11E; A11G¸ A11J; A12AX; B03AD and B03AE). Categories were defined similarly to polypharmacy, i.e. 0–4, ≥5 and ≥10 active substances.

## Results

### Selection procedure and characteristics of participants

Of the 5064 participants at follow-up, 126 (2.5%) were excluded due to missing data for socio-economic characteristics or body mass index, leaving 4938 participants (97.5%) for the current analysis. The characteristics of the included and excluded participants are summarized in Additional file 1 **:** Table S2. Excluded participants lived more frequently alone, while no differences were found for the other individual and socio-economic characteristics.

### Prevalence of polypharmacy

Prevalence of any drug use, polypharmacy and excessive polypharmacy are summarized in Table 1. Almost six out of ten (59.7%) participants reported taking at least one drug; one out of nine (11.8%) was with polypharmacy, and slightly over 1 % (1.4%) was on excessive polypharmacy (Table 1). Cardiovascular drugs were the most frequent, being prescribed to over one third (37.3%) of the participants; psychiatric drugs ranked second highest, being prescribed to one sixth (15.8%) of the participants. Among cardiovascular drugs, the two most frequently prescribed categories were antihypertensive and hypolipidemic drugs (Table 1).

**Table 1:** Prevalence of polypharmacy and of the main drugs prescribed, Colaus study, Switzerland, 2009–2012, 4938 participants

	Frequency n (%)	95% CI
Any drug	2947 (59.7)	(58.3–61.1)
Polypharmacy (≥5 drugs)	580 (11.8)	(10.9–12.7)
Excessive polypharmacy (≥10 drugs)	69 (1.4)	(1.1–1.8)
Cardiovascular	1843 (37.3)	(36.0–38.7)
Antihypertensive drugs	1327 (26.9)	(25.6–28.1)
Angiotensin receptor blockers	644 (13.0)	(12.1–14.0)
Beta-blockers	444 (9.0)	(8.2–9.8)
Angiotensin converting enzyme inhibitors	350 (7.1)	(6.4–7.8)
Calcium channel blockers	229 (4.6)	(4.1–5.3)
Diuretics	158 (3.2)	(2.7–3.7)
Other	81 (1.6)	(1.3–2.0)
Hypolipidemic drugs	1029 (20.8)	(19.7–22.0)
Statins	861 (17.4)	(16.4–18.5)
Other hypolipidemic drugs	219 (4.4)	(3.9–5.0)
Antiplatelet drugs	572 (11.6)	(10.7–12.5)
Aspirin	527 (10.7)	(9.8–11.6)
Vitamin K antagonists	108 (2.2)	(1.8–2.6)
Psychiatric	781 (15.8)	(14.8–16.9)
Antidepressants	516 (10.5)	(9.6–11.3)
Anxiolytics	243 (4.9)	(4.3–5.6)
Hypnotics and sedatives	230 (4.7)	(4.1–5.3)
Antipsychotics	54 (1.1)	(0.8–1.4)
Analgesics	657 (13.3)	(12.4–14.3)
Anilides	110 (2.2)	(1.8–2.7)
Non-steroidal anti-inflammatory drugs	447 (9.1)	(8.3–9.9)
Opioids	61 (1.2)	(0.9–1.6)
Vitamins and minerals	616 (12.5)	(11.6–13.4)
Gastro-intestinal Drugs	469 (9.5)	(8.7–10.3)
Antiacids	351 (7.1)	(6.4–7.9)
Drugs for constipation	68 (1.4)	(1.1–1.7)
Other^a^	114 (2.3)	(1.9–2.8)
Antidiabetic drugs	274 (5.6)	(4.9–6.2)
Oral antidiabetics	252 (5.1)	(4.5–5.8)
Insulin	59 (1.2)	(0.9–1.5)

^a^ATC codes A01, A03, A04, A05, A07 and A09. Results are expressed as number of participants (percentage) and as 95% confidence interval (CI)

### Determinants of polypharmacy

**Fig. 1:**
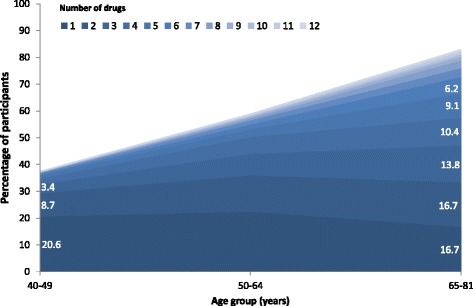
Frequency of drug intake according to age group, in the 4938 participants of the CoLaus study, Switzerland

The distribution of the number of drugs consumed according to age groups is indicated in Fig. 1; the older the participant, the more drugs he/she consumed, with a considerable increase in the prevalence of polypharmacy.

The bivariate and multivariate analyses of the individual and socioeconomic determinants of polypharmacy are summarized in Table 2. Participants with polypharmacy were older, had a higher BMI, were less well educated, and were more frequently former smokers. These findings were further confirmed by multivariate analysis including all determinants simultaneously (Table 2). Conversely, no association was found between polypharmacy and gender, marital status or country of birth (Table 2). Similar findings were obtained for participants reporting excessive polypharmacy, although the association with smoking was no longer significant (Additional file 1 **:** Table S3).

**Table 2:** Bivariate and multivariate analysis of the factors associated with polypharmacy (≥5 different drugs/day), Colaus study, Switzerland, 2009–2012, 4938 participants

	No (*n* = 4358)	Yes (*n* = 580)	*P*-value	Multivariate	P-value for trend
Gender			0.973		
Woman	2325 (53.4)	309 (53.3)		1 (ref.)	
Man	2033 (46.7)	271 (46.7)		0.92 (0.75–1.12)	
Age (years)	56.6 ± 10.1	66.0 ± 9.1	<0.001		
Age group (%)			<0.001		<0.001
40–49	1359 (31.2)	40 (6.9)		1 (ref.)	
50–64	1970 (45.2)	187 (32.2)		2.90 (2.04–4.12)	
65–81	1029 (23.6)	353 (60.9)		10.3 (7.26–14.5)	
BMI categories (%)			<0.001		<0.001
Normal + underweight	2034 (46.7)	125 (21.6)		1 (ref.)	
Overweight	1685 (38.7)	247 (42.6)		2.09 (1.65–2.66)	
Obese	639 (14.7)	208 (35.9)		4.38 (3.39–5.66)	
Education (%)			<0.001		0.002
High	995 (22.8)	70 (12.1)		1 (ref.)	
Middle	1154 (26.5)	124 (21.4)		1.15 (0.83–1.58)	
Low	2209 (50.7)	386 (66.6)		1.56 (1.17–2.07)	
Marital status (%)			0.121		
Living alone	1851 (42.5)	266 (45.9)		1 (ref.)	
Living in a couple	2507 (57.5)	314 (54.1)		0.86 (0.71–1.05)	
Born in Switzerland (%)			0.303		
No	1621 (37.2)	203 (35.0)		1 (ref.)	
Yes	2737 (62.8)	377 (65.0)		0.87 (0.72–1.06)	
Smoking status (%)			<0.001		<0.001
Never	1816 (41.7)	192 (33.1)		1 (ref.)	
Former	1588 (36.4)	271 (46.7)		1.42 (1.14–1.75)	
Current	954 (21.9)	117 (20.2)		1.63 (1.25–2.12)	

*BMI* Body mass index. Bivariate analysis using chi-square for categorical variables and student’s t-test for continuous variables; results are expressed as number of participants (column percentage) or as mean ± standard deviation. Multivariate analysis using logistic regression; results are expressed as odds ratio and (95% confidence interval)

### Sensitivity analysis

When OTC drugs were considered, the prevalence rates of polypharmacy and excessive polypharmacy were 14.7% and 1.8%, respectively. The results of the multivariate analysis are provided in Additional file 1 **:** Table S4 and Additional file 1 **:** Table S5. Similar associations were found as for the original analysis; further, men had a lower likelihood of being on polypharmacy (Additional file 1 **:** Table S4), while no gender differences were found for excessive polypharmacy (Additional file 1 **:** Table S5).

When all active substances were considered (including active substances in OTC drugs), the prevalence rate of taking ≥5 active substances/day was 16.9% and of taking ≥10 active substances/day was 2.3%. The results of the multivariate analysis using active substances are provided in Additional file 1 **:** Table S6 and Additional file 1 **:** Table S7. Results were similar to the previous sensitivity analysis; further, living in a couple was associated with a lower likelihood of taking ≥10 active substances/day (Additional file 1 **:** Table S7).

## Discussion

There are few studies on the prevalence and the determinants of polypharmacy and excessive polypharmacy in the Swiss mid-aged population [12, 28]. Our results show that one out of nine (11.8%) participants aged between 40 and 81 is on polypharmacy, but that less than two out of one hundred (1.4%) are on excessive polypharmacy. Increasing age, body mass index, and lower education independently increase the likelihood of being with polypharmacy or on excessive polypharmacy; a positive association between smoking and polypharmacy was also found.

### Prevalence of polypharmacy

The overall prevalence of polypharmacy was 11.8%, and it increased considerably with age, from 2.9% for age group 40–49 to 25.5% for age group 65–81. Comparison with the literature is difficult as there are differences on how data were collected, in the age groups and, definitions of polypharmacy [29]. In Switzerland, a study based on claims from the largest health insurance reported a prevalence of polypharmacy of 16.7% among adults, and of 41.2% in individuals aged ≥65 years [12]. The prevalence was based on health claims, and only subjects who asked for reimbursement of at least one drug were included. The other Swiss study was based on patients followed in a university primary care setting and reported prevalence rates ranging between 20.8% for age group 50–54 and 54.6% for age group 75–80 [28]. A Scottish study based on electronic data from pharmacy claims reported an overall prevalence of polypharmacy of 22.1% in individuals aged ≥20 years [3], while an Irish study using the same methodology reported a prevalence of 21.5% in individuals aged ≥20 years; 30.2% for age group [45–64] and 60.4% in individuals aged ≥65 years [11]. A possible explanation for these higher values is that individuals consuming “occasional” drugs such as antibiotics or anti-histaminics were also included. Similarly, in another Scottish study, the prevalence of polypharmacy was 36% for age group [60–70]; this higher prevalence could be due to a different definition of polypharmacy, i.e. ≥4 instead of ≥5 drugs [13]. Overall, our results indicate that prevalence of polypharmacy is common in the Swiss mid-aged population, and that almost one quarter of individuals aged 65–81 years are with polypharmacy.

### Determinants of polypharmacy

Increased age was associated with increased polypharmacy and excessive polypharmacy, a finding consistently reported in the literature [1, 2, 11–13]. This reflects the increase in the number of pathologies requiring therapy with ageing, or the difficulty to stop treatment once it has been initiated, leading to cumulative prescriptions. Still, the fact that four out of ten participants aged 65–81 were with polypharmacy stresses the need for the optimization of the prescriptions, such as using for example of the START/STOPP criteria [30], the adoption of a patient-centered rather than a disease-centered approach, and an effective physician-patient communication [31].

Increased body mass index was associated with increased polypharmacy and excessive polypharmacy rates, a finding also in agreement with the literature [4, 32]. A likely explanation is the wide array of comorbidities associated with obesity, namely diabetes, hypertension, dyslipidemia and arthrosis, which frequently require multiple treatments [33].

Participants with a lower education level presented higher rates of polypharmacy. Such association has already been reported, but mostly among elderly subjects [6, 10, 16]. An explanation is that subjects with a lower education level tend to present higher multimorbidity rates [8, 9], possibly due to adverse socio-economic conditions or to less interest for preventive measures. Still, our results suggest that even among middle aged subjects, a low educational level leads to increased rates of polypharmacy.

Smoking was associated with a higher prevalence of polypharmacy, a finding in accordance with another study [4]. Possible explanations include the higher prevalence of pulmonary, cancer and psychiatric disease among smokers, leading to the use of more drugs. Unfortunately, due the scope of the CoLaus study, detailed information regarding those diseases is not available and it would be of interest that this analysis be replicated.

### Classes of drugs

Cardiovascular drugs were the most prescribed therapeutic class, a finding in agreement with the literature [2, 11, 34]. Antihypertensives ranked first, closely followed by statins. Possible explanations include the relatively high prevalence of cardiovascular risk factors in this population [35, 36] and the existence of guidelines regarding cardiovascular risk factor management [37–39]. Conversely, the high prevalence of psychiatric drugs (namely anxiolytics, hypnotics and sedatives) and of analgesics raises some concerns, as these drugs are not supposed to be prescribed on a long term basis.

### Sensitivity analysis

Considering only the number of drugs prescribed might underestimate the prevalence of polypharmacy, as subjects can acquire other products over the counter. Considering also non-prescribed drugs increased the prevalence of polypharmacy from 11.8% to 14.7%, but had no effect on its determinants.

Further, as drugs might contain combinations of active substances, simply counting the number of drugs (including OTC) might still underestimate the prevalence rate of polypharmacy. Indeed, when all active substances were considered, the prevalence of participants taking ≥5 active substances was 5.1% higher than the rate based on drugs (0.9% higher if at least 10 active substances). Again, no significant changes were found regarding the main determinants of taking ≥5 active substances. Overall, our results suggest that the definition of polypharmacy (only prescribed drugs, prescribed + OTC or active substances) considerably impacts the prevalence rates but does not influence significantly its determinants. Studies assessing both polypharmacy and active substances in the general population are scarce and it would be of interest that our results be replicated in other settings.

### Clinical implications

With the ageing of the population, the number of individuals with polypharmacy will increase. This will require general practitioners, hospital medical staff and pharmacists to be increasingly attentive to such a condition, in order to prevent over-prescription and the occurrence of drug-drug interactions and adverse drug reactions. In the forthcoming years, drug prescriptors and dispensers will be required to optimize prescriptions, a difficult task where the pros of adding an extra drug and the cons related to its possible adverse effects will have to be carefully balanced. Hence, it would be of interest that strategies aimed at optimizing polypharmacy are provided to health professionals either at the pre or at the postgraduate level. For instance, in Switzerland, there are initiatives aimed at reducing inappropriate polypharmacy that provide medication reconciliation to ambulatory patients [40]. Indeed, such strategies have been shown to reduce polypharmacy and its potential drug-drug interactions [41] and to be cost-saving [42]. Similarly, the use of medicines combining several drugs may tackle the treatment burden and improve adherence [43].

### Strengths and limitations

This study has several strengths. Firstly, it was based on a large mid-aged population-based sample, allowing to estimate the prevalence of polypharmacy at the population level including non-prescribed (OTC) medicines, a condition that studies based on pharmacy claims cannot perform in Switzerland [3, 12]. Secondly, it clearly assessed drugs taken on a regular basis, precluding a possible overestimation bias due to the occasional consumption of drugs. Finally, and contrary to most studies [1–3, 6, 10–12], several definitions for polypharmacy were applied, allowing a wider comparison with the existing literature [22].

This study has also some limitations. First, participation rate was low (41%), but in line with other epidemiological studies [44]. Thus, a recruitment bias cannot be excluded, the healthiest participants being selected, which would underestimate the prevalence rates of polypharmacy and excessive polypharmacy. Still, the distribution of age groups in our sample was comparable to the source population and there was no difference in gender distribution between the source population and the CoLaus participants (not shown). Further, our results provide a conservative estimate for the prevalence rates of polypharmacy and excessive polypharmacy, and the fact that 25.6% of participants aged 65–81 were with polypharmacy (and 2.7% on excessive polypharmacy) is already concerning. Secondly, it was not possible to assess all comorbidities in our participants; hence, it was not possible to assess if (excessive) polypharmacy was due to increased number of comorbidities. Third, due to legal constraints, it was not possible to cross-check the information provided by the participants with data from medical or pharmaceutical electronic records. No specific training regarding drug collection was provided to the research assistants. Complete medication reconciliation or tools such as the Swiss polymedication check or the brown bags’ method are difficult to apply in large samples due to economic and human resources issues [40, 45, 46]. However, recent studies suggest that self-reported information on medication use closely relates with pharmacy records [47]; hence, memory bias might be small and the impact on prevalence rates might be reduced.

## Conclusion

In a Swiss mid-aged population-based sample, at least one out of nine participants of our sample is on polypharmacy. Age, body mass index, smoking and lower education independently increase the likelihood of being on polypharmacy.

## Additional file

Additional file 1: Table S1. ATC codes of drugs combining active substances. **Table S2.** Characteristics of participants included and excluded from the study. **Table S3.** Bivariate and multivariate analysis of the factors associated with excessive polypharmacy (≥10 different drugs/day), Colaus study, Switzerland, 2009–2012. **Table S4.** Bivariate and multivariate analysis of the factors associated with polypharmacy (including OTC drugs) (≥5 different drugs/day), Colaus study, Switzerland, 2009–2012, 4938 participants. **Table S5.** Bivariate and multivariate analysis of the factors associated with polypharmacy (including OTC drugs) (≥10 different drugs/day), Colaus study, Switzerland, 2009–2012, 4938 participants. **Table S6.** Bivariate and multivariate analysis of the factors associated with taking ≥5 different pharmacologically active substances/day, Colaus study, Switzerland, 2009–2012, 4938 participants. **Table S7.** Bivariate and multivariate analysis of the factors associated with taking ≥10 different pharmacologically active substances/day, Colaus study, Switzerland, 2009–2012, 4938 participants. (DOCX 51 kb)

## Abbreviations

BMI: Body Mass Index
CVD: Cardiovascular diseases
FU: Follow-up
OTC: Over the counter
