# Supplementary material for: Prevalence and determinants of polypharmacy in Switzerland: data from the CoLaus study
Source: BMC Health Serv Res. 2017 Dec 21;17:840. doi: 10.1186/s12913-017-2793-z (PMC5740765; doi:10.1186/s12913-017-2793-z)
Supplement: Additional file 1: Table S1. — ATC codes of drugs combining active substances. Table S2. Characteristics of participants included and excluded from the study. Table S3. Bivariate and multivariate analysis of the factors associated with excessive polypharmacy (≥10 different drugs/day), Colaus study, Switzerland, 2009–2012. Table S4. Bivariate and multivariate analysis of the factors associated with polypharmacy (including OTC drugs) (≥5 different drugs/day), Colaus study, Switzerland, 2009–2012, 4938 participants. Table S5. Bivariate and multivariate analysis of the factors associated with polypharmacy (including OTC drugs) (≥10 different drugs/day), Colaus study, Switzerland, 2009–2012, 4938 participants. Table S6. Bivariate and multivariate analysis of the factors associated with taking ≥5 different pharmacologically active substances/day, Colaus study, Switzerland, 2009–2012, 4938 participants. Table S7. Bivariate and multivariate analysis of the factors associated with taking ≥10 different pharmacologically active substances/day, Colaus study, Switzerland, 2009–2012, 4938 participants. (DOCX 51 kb) [file 12913_2017_2793_MOESM1_ESM.docx]

**Supplementary 1:** ATC codes of drugs combining active substances.

| **ATC code** | | **Designation** |  |
| --- | --- | --- | --- |
| C01AA52 | [acetyldigoxin, combinations](http://www.whocc.no/atc_ddd_index/?code=C01AA52) | | |
| C01AB51 | [proscillaridin, combinations](http://www.whocc.no/atc_ddd_index/?code=C01AB51) | | |
| C01BA51 | [quinidine, combinations excl. psycholeptics](http://www.whocc.no/atc_ddd_index/?code=C01BA51) | | |
| C01BA71 | [quinidine, combinations with psycholeptics](http://www.whocc.no/atc_ddd_index/?code=C01BA71) | | |
| C01CA30 | [combinations](http://www.whocc.no/atc_ddd_index/?code=C01CA30) | | |
| C01CA51 | [etilefrine, combinations](http://www.whocc.no/atc_ddd_index/?code=C01CA51) | | |
| C01DA20 | [organic nitrates in combination](http://www.whocc.no/atc_ddd_index/?code=C01DA20) | | |
| C01DA52 | [glyceryl trinitrate, combinations](http://www.whocc.no/atc_ddd_index/?code=C01DA52) | | |
| C01DA54 | [methylpropylpropanediol dinitrate, combinations](http://www.whocc.no/atc_ddd_index/?code=C01DA54) | | |
| C01DA55 | [pentaerithrityl tetranitrate, combinations](http://www.whocc.no/atc_ddd_index/?code=C01DA55) | | |
| C01DA57 | [propatylnitrate, combinations](http://www.whocc.no/atc_ddd_index/?code=C01DA57) | | |
| C01DA58 | [isosorbide dinitrate, combinations](http://www.whocc.no/atc_ddd_index/?code=C01DA58) | | |
| C01DA59 | [trolnitrate, combinations](http://www.whocc.no/atc_ddd_index/?code=C01DA59) | | |
| C01DA63 | [eritrityl tetranitrate, combinations](http://www.whocc.no/atc_ddd_index/?code=C01DA63) | | |
| C01DA70 | [organic nitrates in combination with psycholeptics](http://www.whocc.no/atc_ddd_index/?code=C01DA70) | | |
| C01DX51 | [itramin tosilate, combinations](http://www.whocc.no/atc_ddd_index/?code=C01DX51) | | |
| C01DX52 | [prenylamine, combinations](http://www.whocc.no/atc_ddd_index/?code=C01DX52) | | |
| C01DX53 | [oxyfedrine, combinations](http://www.whocc.no/atc_ddd_index/?code=C01DX53) | | |
| C01DX54 | [benziodarone, combinations](http://www.whocc.no/atc_ddd_index/?code=C01DX54) | | |
| C02AA03 | [combinations of rauwolfia alkaloids](http://www.whocc.no/atc_ddd_index/?code=C02AA03) | | |
| C02AA52 | [reserpine, combinations](http://www.whocc.no/atc_ddd_index/?code=C02AA52) | | |
| C02AA53 | [combinations of rauwolfia alkoloids, combinations](http://www.whocc.no/atc_ddd_index/?code=C02AA53) | | |
| C02AA57 | [bietaserpine, combinations](http://www.whocc.no/atc_ddd_index/?code=C02AA57) | | |
| C03BA82 | [clorexolone, combinations with psycholeptics](http://www.whocc.no/atc_ddd_index/?code=C03BA82) | | |
| C04AE51 | [ergoloid mesylates, combinations](http://www.whocc.no/atc_ddd_index/?code=C04AE51) | | |
| C04AE54 | [dihydroergocristine, combinations](http://www.whocc.no/atc_ddd_index/?code=C04AE54) | | |
| C05AX02 | [bismuth preparations, combinations](http://www.whocc.no/atc_ddd_index/?code=C05AX02) | | |
| C05AX03 | [other preparations, combinations](http://www.whocc.no/atc_ddd_index/?code=C05AX03) | | |
| C05BA51 | [heparinoid, combinations](http://www.whocc.no/atc_ddd_index/?code=C05BA51) | | |
| C05BA53 | [heparin, combinations](http://www.whocc.no/atc_ddd_index/?code=C05BA53) | | |
| C05BB56 | [glucose, combinations](http://www.whocc.no/atc_ddd_index/?code=C05BB56) | | |
| C05BX51 | [calcium dobesilate, combinations](http://www.whocc.no/atc_ddd_index/?code=C05BX51) | | |
| C05CA51 | [rutoside, combinations](http://www.whocc.no/atc_ddd_index/?code=C05CA51) | | |
| C05CA53 | [diosmin, combinations](http://www.whocc.no/atc_ddd_index/?code=C05CA53) | | |
| C05CA54 | [troxerutin, combinations](http://www.whocc.no/atc_ddd_index/?code=C05CA54) | | |
| C07AA57 | [sotalol, combinations](http://www.whocc.no/atc_ddd_index/?code=C07AA57) | | |
| C07AB52 | [metoprolol, combinations](http://www.whocc.no/atc_ddd_index/?code=C07AB52) | | |
| C07AB57 | [bisoprolol, combinations](http://www.whocc.no/atc_ddd_index/?code=C07AB57) | | |
| C07BA68 | [metipranolol and thiazides, combinations](http://www.whocc.no/atc_ddd_index/?code=C07BA68) | | |
| C07BB52 | [metoprolol and thiazides, combinations](http://www.whocc.no/atc_ddd_index/?code=C07BB52) | | |
| C07CB53 | [atenolol and other diuretics, combinations](http://www.whocc.no/atc_ddd_index/?code=C07CB53) | | |
| C08CA55 | [nifedipine, combinations](http://www.whocc.no/atc_ddd_index/?code=C08CA55) | | |
| C08DA51 | [verapamil, combinations](http://www.whocc.no/atc_ddd_index/?code=C08DA51) | | |
| C10AD52 | [nicotinic acid, combinations](http://www.whocc.no/atc_ddd_index/?code=C10AD52) | | |

**Supplementary table 2**: characteristics of participants included and excluded from the study

|  | Included | Excluded | P-value |
| --- | --- | --- | --- |
| Sample size | 4938 | 126 |  |
| Women (%) | 2634 (53.3) | 73 (57.9) | 0.307 |
| Age (years) | 57.7 ± 10.5 | 60.1 ± 12.2 | 0.010 |
| Age group (%) |  |  | 0.004 |
| [35-50[ | 1399 (28.3) | 32 (25.4) |  |
| [50-65[ | 2157 (43.7) | 42 (33.3) |  |
| [65+ | 1382 (28.0) | 52 (41.3) |  |
| BMI categories (%) |  |  | 0.346 |
| Normal + underweight | 2159 (43.7) | 21 (34.4) |  |
| Overweight | 1932 (39.1) | 28 (45.9) |  |
| Obese | 847 (17.2) | 12 (19.7) |  |
| Education (%) |  |  | 0.009 |
| High | 1065 (21.6) | 14 (11.6) |  |
| Middle | 1278 (25.9) | 28 (23.1) |  |
| Low | 2595 (52.6) | 79 (65.3) |  |
| Living alone (%) | 2117 (42.9) | 85 (67.5) | <0.001 |
| Born in Switzerland (%) | 3114 (63.1) | 70 (55.6) | 0.085 |
| Smoking status (%) |  |  | 0.673 |
| Never | 2008 (40.7) | 27 (39.1) |  |
| Former | 1859 (37.7) | 24 (34.8) |  |
| Current | 1071 (21.7) | 18 (26.1) |  |

BMI, body mass index. Results are expressed as number of participants (column percentage) or as mean ± standard deviation. Between-group comparisons using chi-square for categorical variables and student’s t-test for continuous variables.

**Supplementary table 3**: bivariate and multivariate analysis of the factors associated with excessive polypharmacy (≥10 different drugs/day), Colaus study, Switzerland, 2009-2012.

|  | Excessive polypharmacy | |  |  |  |
| --- | --- | --- | --- | --- | --- |
|  | **No (n=4869)** | **Yes (n=69)** | **P-value** | **Multivariate analysis** | **P-value for trend** |
| Gender |  |  | 0.355 |  |  |
| Woman | 2601 (53.4) | 33 (47.8) |  | 1 (ref.) |  |
| Man | 2268 (46.6) | 36 (52.2) |  | 1.23 (0.74 - 2.05) |  |
| Age (years) | 57.6 ± 10.4 | 65.0 ± 9.7 | <0.001 |  |  |
| Age group (%) |  |  | <0.001 |  | <0.001 |
| 40-49 | 1392 (28.6) | 7 (10.1) |  | 1 (ref.) |  |
| 50-64 | 2132 (43.8) | 25 (36.2) |  | 2.07 (0.89 - 4.83) |  |
| 65-81 | 1345 (27.6) | 37 (53.6) |  | 4.49 (1.95 - 10.3) |  |
| BMI categories (%) |  |  | <0.001 |  | <0.001 |
| Normal + underweight | 2147 (44.1) | 12 (17.4) |  | 1 (ref.) |  |
| Overweight | 1906 (39.2) | 26 (37.7) |  | 2.01 (1.00 - 4.07) |  |
| Obese | 816 (16.8) | 31 (44.9) |  | 5.01 (2.51 - 10.0) |  |
| Education (%) |  |  | 0.002 |  | 0.015 |
| High | 1062 (21.8) | 3 (4.4) |  | 1 (ref.) |  |
| Middle | 1258 (25.8) | 20 (29.0) |  | 4.58 (1.35 - 15.6) |  |
| Low | 2549 (52.4) | 46 (66.7) |  | 4.36 (1.33 - 14.2) |  |
| Marital status (%) |  |  | 0.184 |  |  |
| Living alone | 2082 (42.8) | 35 (50.7) |  | 1 (ref.) |  |
| Living in a couple | 2787 (57.2) | 34 (49.3) |  | 0.68 (0.42 - 1.12) |  |
| Born in Switzerland (%) |  |  | 0.528 |  |  |
| No | 1796 (36.9) | 28 (40.6) |  | 1 (ref.) |  |
| Yes | 3073 (63.1) | 41 (59.4) |  | 0.73 (0.45 - 1.20) |  |
| Smoking status (%) |  |  | 0.114 |  | 0.104 |
| Never | 1988 (40.8) | 20 (29.0) |  | 1 (ref.) |  |
| Former | 1826 (37.5) | 33 (47.8) |  | 1.47 (0.83 - 2.62) |  |
| Current | 1055 (21.7) | 16 (23.2) |  | 1.76 (0.89 - 3.50) |  |

BMI, body mass index. Bivariate analysis using chi-square for categorical variables and student’s t-test for continuous variables; results are expressed as number of participants (column percentage) or as mean ± standard deviation. Multivariate analysis using logistic regression; results are expressed as odds ratio and (95% confidence interval).

**Supplementary table 4:** bivariate and multivariate analysis of the factors associated with polypharmacy (including OTC drugs) (≥5 different drugs/day), Colaus study, Switzerland, 2009-2012, 4938 participants

|  | No (n=4211) | Yes (n=727) | P-value | Multivariate | P-value for trend |
| --- | --- | --- | --- | --- | --- |
| Gender |  |  | 0.002 |  |  |
| Woman | 2208 (52.4) | 426 (58.6) |  | 1 (ref.) |  |
| Man | 2003 (47.6) | 301 (41.4) |  | 0.71 (0.59 - 0.85) |  |
| Age (years) | 56.4 ± 10.1 | 65.2 ± 9.3 | <0.001 |  |  |
| Age group (%) |  |  | <0.001 |  | <0.001 |
| 40-49 | 1343 (31.9) | 56 (7.7) |  | 1 (ref.) |  |
| 50-64 | 1897 (45.1) | 260 (35.8) |  | 3.00 (2.22 - 4.05) |  |
| 65-81 | 971 (23.1) | 411 (56.5) |  | 9.04 (6.7 - 12.2) |  |
| BMI categories (%) |  |  | <0.001 |  | <0.001 |
| Normal +underweight | 1961 (46.6) | 198 (27.2) |  | 1 (ref.) |  |
| Overweight | 1632 (38.8) | 300 (41.3) |  | 1.71 (1.39 - 2.09) |  |
| Obese | 618 (14.7) | 229 (31.5) |  | 3.21 (2.56 - 4.04) |  |
| Education (%) |  |  | <0.001 |  | 0.004 |
| High | 973 (23.1) | 92 (12.7) |  | 1 (ref.) |  |
| Middle | 1104 (26.2) | 174 (23.9) |  | 1.27 (0.96 - 1.68) |  |
| Low | 2134 (50.7) | 461 (63.4) |  | 1.44 (1.12 - 1.86) |  |
| Marital status (%) |  |  | 0.040 |  |  |
| Living alone | 1780 (42.3) | 337 (46.4) |  | 1 (ref.) |  |
| Living in a couple | 2431 (57.7) | 390 (53.7) |  | 0.89 (0.75 - 1.06) |  |
| Born in Switzerland (%) |  |  | 0.123 |  |  |
| No | 1574 (37.4) | 250 (34.4) |  | 1 (ref.) |  |
| Yes | 2637 (62.6) | 477 (65.6) |  | 0.93 (0.77 - 1.11) |  |
| Smoking status (%) |  |  | <0.001 |  | <0.001 |
| Never | 1758 (41.8) | 250 (34.4) |  | 1 (ref.) |  |
| Former | 1534 (36.4) | 325 (44.7) |  | 1.38 (1.14 - 1.67) |  |
| Current | 919 (21.8) | 152 (20.9) |  | 1.61 (1.27 - 2.03) |  |

BMI, body mass index. Bivariate analysis using chi-square for categorical variables and student’s t-test for continuous variables; results are expressed as number of participants (column percentage) or as mean ± standard deviation. Multivariate analysis using logistic regression; results are expressed as odds ratio and (95% confidence interval).

**Supplementary table 5:** bivariate and multivariate analysis of the factors associated with polypharmacy (including OTC drugs) (≥10 different drugs/day), Colaus study, Switzerland, 2009-2012, 4938 participants

|  | No (n=4851) | | Yes (n=87) | | P-value | | Multivariate | P-value for trend |
| --- | --- | --- | --- | --- | --- | --- | --- | --- |
| Gender | |  |  | 0.602 | | |  |  |
| Woman | | 2590 (53.4) | 44 (50.6) |  | | | 1 (ref.) |  |
| Man | | 2261 (46.6) | 43 (49.4) |  | | | 1.115 (0.708 - 1.758) |  |
| Age (years) | | 57.6 ± 10.4 | 65.7 ± 9.2 | <0.001 | | |  |  |
| Age group (%) | |  |  | <0.001 | | |  | <0.001 |
| 40-49 | | 1392 (28.7) | 7 (8.1) |  | | | 1 (ref.) |  |
| 50-64 | | 2127 (43.9) | 30 (34.5) |  | | | 2.516 (1.096 - 5.772) |  |
| 65-81 | | 1332 (27.5) | 50 (57.5) |  | | | 6.286 (2.79 - 14.167) |  |
| BMI categories (%) | |  |  | <0.001 | | |  | <0.001 |
| Normal +underweight | | 2141 (44.1) | 18 (20.7) |  | | | 1 (ref.) |  |
| Overweight | | 1898 (39.1) | 34 (39.1) |  | | | 1.776 (0.984 - 3.207) |  |
| Obese | | 812 (16.7) | 35 (40.2) |  | | | 3.816 (2.105 - 6.918) |  |
| Education (%) | |  |  | 0.002 | | |  | 0.0210 |
| High | | 1059 (21.8) | 6 (6.9) |  | | | 1 (ref.) |  |
| Middle | | 1256 (25.9) | 22 (25.3) |  | | | 2.476 (0.991 - 6.183) |  |
| Low | | 2536 (52.3) | 59 (67.8) |  | | | 2.750 (1.166 - 6.488) |  |
| Marital status (%) | |  |  | 0.213 | | |  |  |
| Living alone | | 2074 (42.8) | 43 (49.4) |  | | 1 (ref.) | |  |
| Living in a couple | | 2777 (57.3) | 44 (50.6) |  | | 0.746 (0.481 - 1.159) | |  |
| Born in Switzerland (%) | |  |  | 0.846 | |  | |  |
| No | | 1791 (36.9) | 33 (37.9) |  | | 1 (ref.) | |  |
| Yes | | 3060 (63.1) | 54 (62.1) |  | | 0.794 (0.507 - 1.243) | |  |
| Smoking status (%) | |  |  | 0.156 | |  | | 0.0793 |
| Never | | 1981 (40.8) | 27 (31) |  | | 1 (ref.) | |  |
| Former | | 1819 (37.5) | 40 (46) |  | | 1.354 (0.816 - 2.247) | |  |
| Current | | 4439 (100) | 582 (100) |  | | 1.717 (0.939 - 3.140) | |  |

BMI, body mass index. Bivariate analysis using chi-square for categorical variables and student’s t-test for continuous variables; results are expressed as number of participants (column percentage) or as mean ± standard deviation. Multivariate analysis using logistic regression; results are expressed as odds ratio and (95% confidence interval).

**Supplementary table 6**: bivariate and multivariate analysis of the factors associated with taking ≥5 different pharmacologically active substances/day, Colaus study, Switzerland, 2009-2012, 4938 participants

|  | | No (n=4102) | Yes (n=836) | P-value | Multivariate | P-value for trend |
| --- | --- | --- | --- | --- | --- | --- |
| Gender |  | |  | 0.002 |  |  |
| Woman | 2147 (52.3) | | 487 (58.3) |  | 1 (ref.) |  |
| Man | 1955 (47.7) | | 349 (41.8) |  | 0.703 (0.593 - 0.834) |  |
| Age (years) | 56.3 ± 10.1 | | 64.7 ± 9.4 | <0.001 |  |  |
| Age group (%) |  | |  | <0.001 |  | <0.001 |
| 40-49 | 1329 (32.4) | | 70 (8.4) |  | 1 (ref.) |  |
| 50-64 | 1848 (45.1) | | 309 (37) |  | 2.882 (2.195 - 3.785) |  |
| 65-81 | 925 (22.6) | | 457 (54.7) |  | 8.253 (6.279 - 10.846) |  |
| BMI categories (%) |  | |  | <0.001 |  | <0.001 |
| Normal +underweight | 1930 (47.1) | | 229 (27.4) |  | 1 (ref.) |  |
| Overweight | 1581 (38.5) | | 351 (42) |  | 1.771 (1.461 - 2.147) |  |
| Obese | 591 (14.4) | | 256 (30.6) |  | 3.225 (2.594 - 4.009) |  |
| Education (%) |  | |  | <0.001 |  | 0.0019 |
| High | 958 (23.4) | | 107 (12.8) |  | 1 (ref.) |  |
| Middle | 1077 (26.3) | | 201 (24) |  | 1.281 (0.983 - 1.669) |  |
| Low | 2067 (50.4) | | 528 (63.2) |  | 1.457 (1.15 - 1.848) |  |
| Marital status (%) |  | |  | 0.050 |  |  |
| Living alone | 1733 (42.3) | | 384 (45.9) |  | 1 (ref.) |  |
| Living in a couple | 2369 (57.8) | | 452 (54.1) |  | 0.905 (0.769 - 1.066) |  |
| Born in Switzerland (%) |  | |  | 0.035 |  |  |
| No | 1542 (37.6) | | 282 (33.7) |  | 1 (ref.) |  |
| Yes | 2560 (62.4) | | 554 (66.3) |  | 0.976 (0.823 - 1.157) |  |
| Smoking status (%) |  | |  | <0.001 |  | <0.001 |
| Never | 1727 (42.1) | | 281 (33.6) |  | 1 (ref.) |  |
| Former | 1479 (36.1) | | 380 (45.5) |  | 1.477 (1.230 - 1.774) |  |
| Current | 896 (21.8) | | 175 (20.9) |  | 1.645 (1.316 - 2.057) |  |

BMI, body mass index. Bivariate analysis using chi-square for categorical variables and student’s t-test for continuous variables; results are expressed as number of participants (column percentage) or as mean ± standard deviation. Multivariate analysis using logistic regression; results are expressed as odds ratio and (95% confidence interval).

**Supplementary table 7**: bivariate and multivariate analysis of the factors associated with taking ≥10 different pharmacologically active substances/day, Colaus study, Switzerland, 2009-2012, 4938 participants

|  | No (n=4825) | Yes (n=113) | P-value | Multivariate | P-value for trend | |
| --- | --- | --- | --- | --- | --- | --- |
| Gender |  |  | 0.958 |  |  | |
| Woman | 2574 (53.4) | 60 (53.1) |  | 1 (ref.) |  | |
| Man | 2251 (46.7) | 53 (46.9) |  | 1.029 (0.688 - 1.539) |  | |
| Age (years) | 57.5 ± 10.4 | 66.1 ± 9.1 | <0.001 |  |  | |
| Age group (%) |  |  | <0.001 |  | <0.001 | |
| 40-49 | 1391 (28.8) | 8 (7.1) |  | 1 (ref.) |  |  |
| 50-64 | 2117 (43.9) | 40 (35.4) |  | 2.88 (1.338 - 6.201) |  | |
| 65-81 | 1317 (27.3) | 65 (57.5) |  | 6.836 (3.22 - 14.511) |  | |
| BMI categories (%) |  |  | <0.001 |  | <0.001 | |
| Normal +underweight | 2135 (44.3) | 24 (21.2) |  | 1 (ref.) |  | |
| Overweight | 1890 (39.2) | 42 (37.2) |  | 1.688 (1.004 - 2.84) |  | |
| Obese | 800 (16.6) | 47 (41.6) |  | 3.943 (2.35 - 6.618) |  | |
| Education (%) |  |  | <0.001 |  | 0.0127 | |
| High | 1057 (21.9) | 8 (7.1) |  | 1 (ref.) |  | |
| Middle | 1249 (25.9) | 29 (25.7) |  | 2.433 (1.097 - 5.396) |  | |
| Low | 2519 (52.2) | 76 (67.3) |  | 2.587 (1.225 - 5.465) |  | |
| Marital status (%) |  |  | 0.026 |  |  | |
| Living alone | 2057 (42.6) | 60 (53.1) |  | 1 (ref.) |  | |
| Living in a couple | 2768 (57.4) | 53 (46.9) |  | 0.644 (0.436 - 0.950) |  | |
| Born in Switzerland (%) |  |  | 0.732 |  |  | |
| No | 1784 (37) | 40 (35.4) |  | 1 (ref.) |  | |
| Yes | 3041 (63) | 73 (64.6) |  | 0.884 (0.592 - 1.321) |  | |
| Smoking status (%) |  |  | 0.039 |  | 0.1348 | |
| Never | 1973 (40.9) | 35 (31) |  | 1 (ref.) |  | |
| Former | 1804 (37.4) | 55 (48.7) |  | 1.47 (0.944 - 2.288) |  | |
| Current | 1048 (21.7) | 23 (20.4) |  | 1.522 (0.878 - 2.641) |  | |

BMI, body mass index. Bivariate analysis using chi-square for categorical variables and student’s t-test for continuous variables; results are expressed as number of participants (column percentage) or as mean ± standard deviation. Multivariate analysis using logistic regression; results are expressed as odds ratio and (95% confidence interval).
